# Application and control of flexible alternating current transmission system devices for voltage stability enhancement of renewable-integrated power grid: A comprehensive review

**DOI:** 10.1016/j.heliyon.2021.e06461

**Published:** 2021-03-11

**Authors:** Bukola Babatunde Adetokun, Christopher Maina Muriithi

**Affiliations:** a Electrical Engineering Department, Pan African University Institute for Basic Sciences, Technology and Innovation, Jomo Kenyatta University of Agriculture and Technology, Nairobi, Kenya; b Department of Electrical and Information Engineering, Landmark University, Omu-aran, Nigeria; c Electrical Engineering Department, Murang'a University of Technology, Murang'a, Kenya

**Keywords:** Control scheme, Grid integration, FACTS devices, Renewable energy, Voltage stability

## Abstract

This paper presents a comprehensive review on the application and control of Flexible Alternating Current Transmission Systems (FACTS) devices in order to improve voltage stability of power grid with high share of renewable energy systems. The rise in the development of renewable energy technologies is driven by the attempts to mitigate the effects of global warming. In addition, global energy demand continues to rise as new electricity-driven systems such as electric vehicles are being developed and as remote locations are being connected to the grid. These changes in the power grid formation comes with attendant challenges which must be addressed. One of these challenges is voltage stability issues. This paper therefore explores the applications of FACTS devices for voltage stability improvement and points out the future research direction in the area of voltage stability enhancement by FACTS devices for modern power grids characterized by increasing levels of renewable energy penetration.

## 1 Introduction

Significant progress is being made on the harnessing of renewable energy (RE) resources to meet the rising global energy demand and also to reduce the influence of climate change and global warming due to excessive exploitation of fossil-fuel [1, 2, 3]. Rapid growth in the design, development, and deployment of Renewable Energy Systems has been on the increase in Europe, China, India, and North America. Kenya also utilises renewable energy sources such as geothermal, wind and hydro, which forms a significant percentage of the total installed generation capacity. Also, the insufficient and erratic power supply in developing countries has raised concerns, with intensified efforts targeted toward the utilization of available RE resources. Thus, the harnessing of RE resources such as solar, wind, hydropower, and geothermal energy has gained global attention [4, 5, 6]. Wind Energy Conversion System (WECS) is one of the most prominent variable RE system. The 2019 annual report of the Global Wind Energy Council (GWEC) states that over 651GW of WECS has been installed globally at the end of 2019. This figure is also projected to reach about 727GW by the end of 2020 [7].

Voltage stability and power quality concerns are the main factors that imposes limitation on the penetration level of renewable energy in transmission systems [8]. In particular, voltage stability becomes the dominant problem to be addressed when the penetration level of RE systems increases significantly [9]. Furthermore, some researchers have analysed the possibilities of a power grid with 100% penetration of renewable energy generation [10, 11, 12, 13, 14, 15, 16, 17]. A detailed and convincing arguments have been put forward in [12], showing that a 100% renewable generation is technically feasible and economically viable. Presently, Iceland has already achieved 100% RE generation. Nations which are near to 100% RE generation are Canada, Brazil, Costa Rica, Uruguay, Norway and Paraguay, which have attained 62%, 76%, 93%, 95%, 97% and 99% respectively [14].

However, in order to achieve technological feasibility and economic viability, ancillary services such as reactive power supports and voltage stability measures must be put in place for the successful integration and operation of variable RE generation [18]. FACTS devices are often employed to enhance power system stability and power quality improvement. There are various types of FACTS devices, each with its attendant features, merits, and demerits [19]. A FACTS device can be used either individually or in coordination with another FACTS device type in order to provide control of transmission system parameters of interest, which are essential to the successful operation of the grid. FACTS devices such as Static Synchronous Compensator (STATCOM), Static VAR Compensator (SVC), Unified Power Flow Controller (UPFC), Static Synchronous Series Compensator (SSSC) and Thyristor-Controlled Series Capacitor (TCSC) are employed in the enhancement of grid voltage stability and power quality with varying degrees of effectiveness. This paper presents a comprehensive review on the current state-of-the-art applications of these devices for voltage stability improvement of RE-integrated power grid. In addition, the features, mode of operation and the attendant merits and demerits of common FACTS devices are provided in this work. Future research focus on the development and application of FACTS devices has also been pointed out.

The rest of this paper is structured as follows: Section 2 deals with the theory of voltage stability and Section 3 presents an overview of FACTS devices. A review of relevant works on the application of FACTS devices for voltage stability enhancement has been provided and examined in Section 4. Section 5 presents a discussion on the future research direction in the area of FACTS device application for voltage stability improvement of power systems with high renewable energy share. This study is concluded in Section 6.

## 2 Voltage stability in power systems

Voltage stability has been defined in [20] as “the ability of a power system to maintain steady voltages at all buses in the system after being subjected to a disturbance from a given initial operating condition”. Conversely, voltage instability is the failure of a power system to maintain steady voltages at its buses after system disturbance has occurred. Voltage instability constitutes a considerable challenge in power systems with inadequate reactive power support to maintain the bus voltages within the required limits. Voltage stability issue is of important concerns when it comes to heavily loaded power systems or increasing system loading. Fault occurrences can also trigger voltage instability in the power network at specific locations within the system or at system level. When voltage instability occurs at system level and there are no immediate remedial control actions, this can result to partial or entire grid collapse.

**Figure 1:**
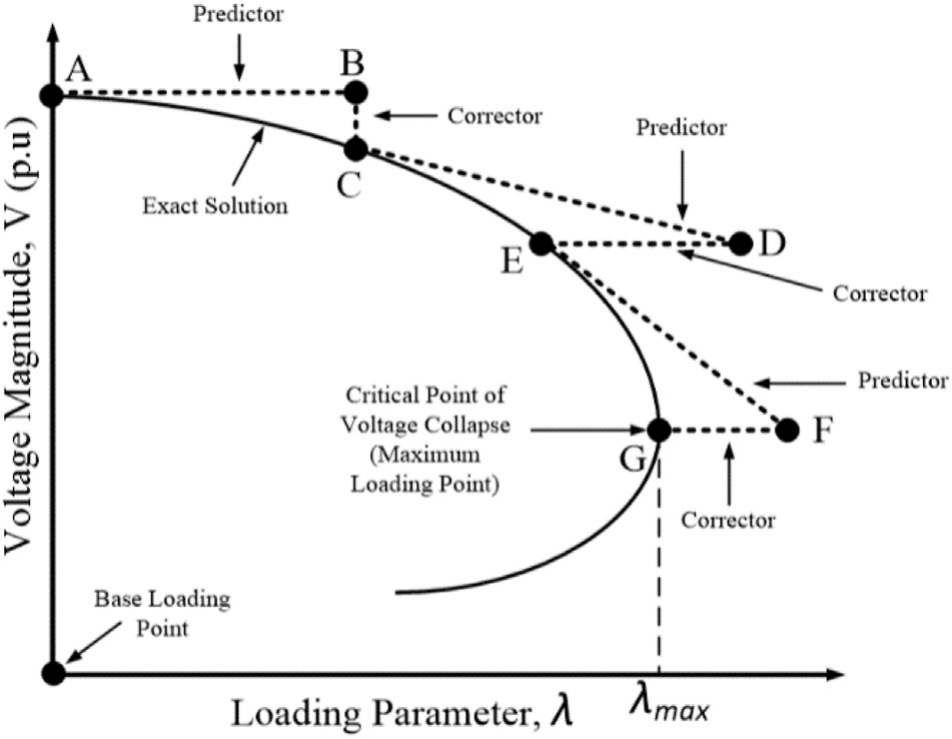
Continuation power flow process.

A well-known method used in the assessment of voltage stability is the continuation power flow (CPF) algorithm. This is an advanced form of the conventional power flow, which is dependent on the Newton Raphson's load flow solution. The CPF is applied when the Jacobian matrix of the load flow equations reaches singularity at point of saddle node bifurcation (SNB). The algorithm is specially employed to generate the active power-voltage (PV) curve, with an incremental change in loading [21]. The CPF also generates the complete curve solution after reaching the voltage collapse point. The SNB point corresponds to the point of maximum loading as indicated in Figure 1.

The CPF algorithm operates on a bifurcation model, whereby a variation in system parameter results in changes in system stability [22]. The CPF utilises the predictor-corrector method as shown in Figure 1 [23].

## 3 Overview of FACTS devices

Original FACTS devices are power electronic-based devices often employed in modern power system to optimize the overall grid performance. FACTS devices can be broadly categorized as either series or shunt. Series FACTS devices include TCSC and SSSC, while STATCOM and SVC are shunt-connected. UPFC is series-shunt connected, thereby combining the advantages of both types. Each FACTS device model can be represented by a set of differential-algebraic equations. This can be generally expressed as [24]:

(1) $$\begin{array}{l} {\dot{x}}_{s}=f\left(x_{s},x_{c},V,\delta \right)\\ {\dot{x}}_{c}=f\left(x_{s},x_{c},V,\delta ,u_{ref}\right)\\ P=g\left(x_{s},x_{c},V,\delta \right)\\ Q=g\left(x_{s},x_{c},V,\delta \right) \end{array}$$

where *x*_*s*_ are the controlled state variables such as thyristor firing angles, *x*_*c*_ are the control system variables, the algebraic variables *V* are the bus voltage amplitudes and *δ* is the phase angles at the buses where the devices are connected. These are vectors in the case of series FACTS devices. The variable *u*_*ref*_ denotes the control inputs, such as reference voltages.

The rest of this section provides an overview of two of these FACTS devices, namely SVC and STATCOM.

### 3.1 Static Var compensator

SVC is a shunt-connected FACTS device, usually made up of controllable Thyristor Switched Capacitors (TSC) and Thyristor Controlled Reactors (TCR). The SVC provides reactive power compensation and control of the terminal bus voltage by controlling the firing angle of the shunt-connected thyristor switch. SVC can be of different configurations, namely:

(a) Thyristor-Controlled-Reactor/Thyristor-Switched-Reactor (TCR/TSR)
(b) Thyristor-Switched-Capacitor (TSC)
(c) Thyristor-Controlled-Reactor-Fixed Capacitor (TCR-FC)
(d) Thyristor-Switched-Capacitor/Thyristor-Controlled-Reactor (TSC-TCR).

**Figure 2:**
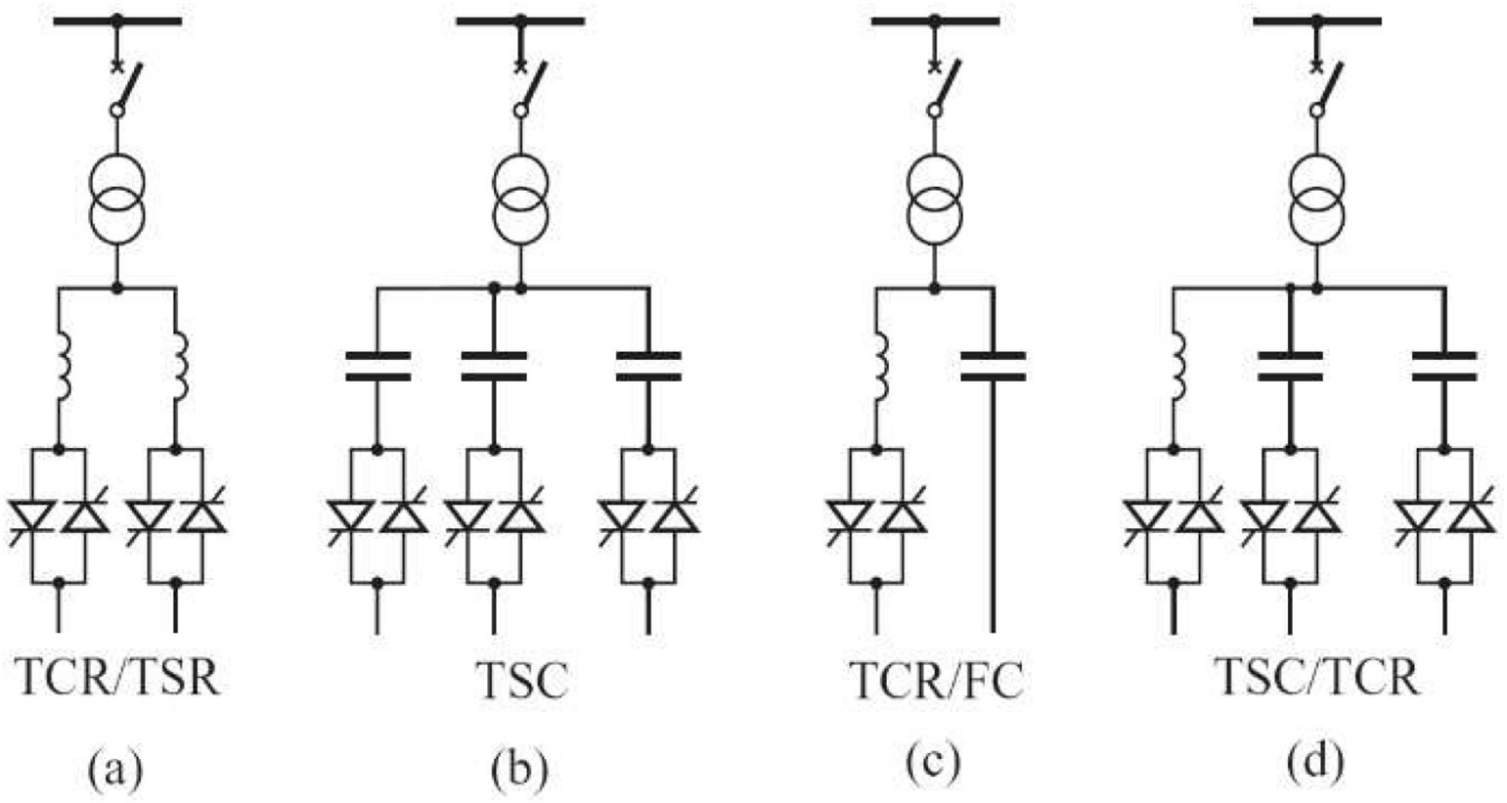
Types of SVC Configuration.

These are illustrated in Figure 2 [25]. For the TSC/TCR type, the back-to-back thyristor switch is connected in series with an inductor and a capacitor. This TSC/TCR combination permits the control of injection and absorption of reactive power.

**Figure 3:**
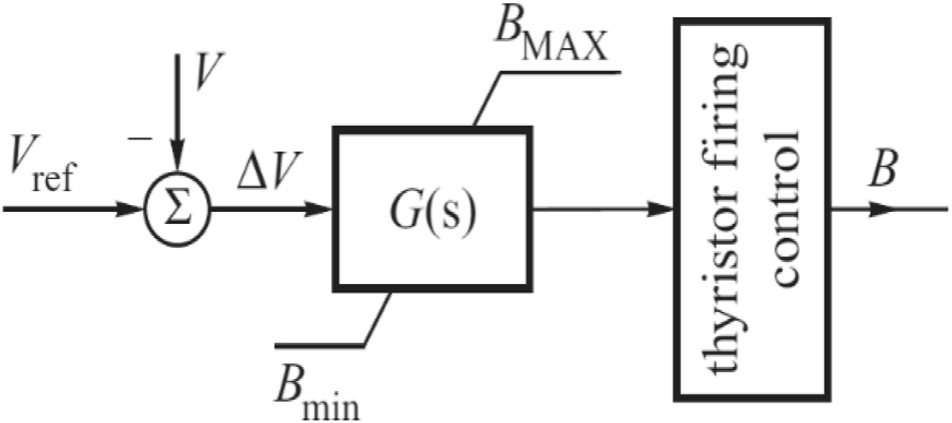
Simplified block diagram of static VAR compensator.

Figure 3 illustrates a simplified block diagram of SVC [25]. The reference voltage (*V*_*ref*_) is the desired voltage rating. The bus voltage (*V*) is measured and compared with *V*_*ref*_. The error difference, Δ*V* is utilized to compute the required firing angle which will keep the bus voltage as close as possible to the *V*_*ref*_*.*

### 3.2 Static Synchronous Compensator

**Figure 4:**
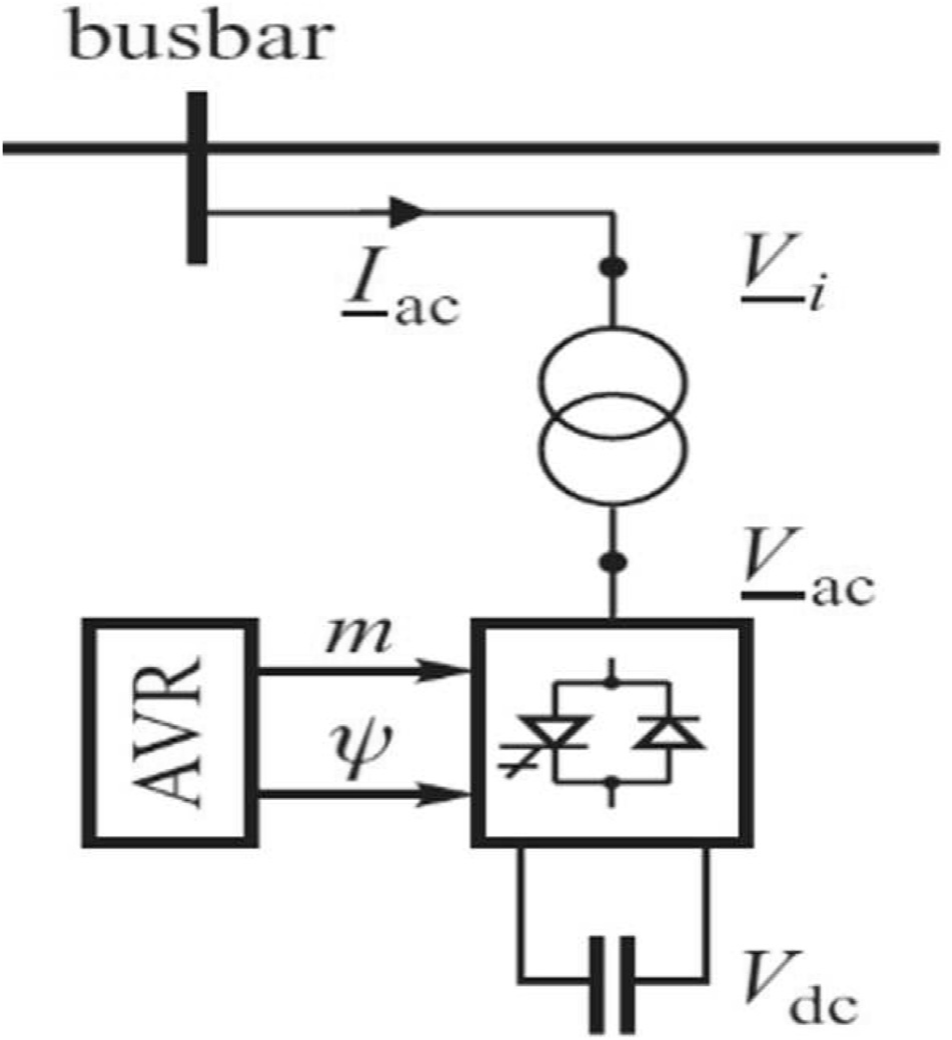
Basic structure of STATCOM.

STATCOM is a shunt-connected FACTS device, which is essentially made up of a Voltage Source Inverter (VSI). The VSI converts the DC input voltage to AC output voltage so as to provide real and reactive power compensation required by the system to which it is connected. The control of reactive power between the converter and the power system is achieved by varying the AC output voltage amplitude. The basic structure of STATCOM is depicted in Figure 4 [25].

Table 1 provides the various features, mode of operation, and the merits and demerits of commonly used FACTS devices for voltage stability enhancement [26, 27, 28, 29, 30].

**Table 1:** Comparisons of FACTS devices.

FACTS	Features	Mode of Operation	Notable Merits and Demerits
TCSC	Series connected capacitor with thyristor-based controller	Controls the total susceptance of the transmission line using the firing angle of the thyristor	• Enhanced real power transfer and better sub-synchronous resonance and oscillation damping. • Requires bulky capacitors and reactors
SSSC	Series-connected, utilises voltage source converter with gate turn-off switches	Compensates the transmission line reactance by means of a current-controlled voltage source	• Does not require bulky capacitors and reactors • Capable of injecting real power by means of an energy source connected to its DC side. • Higher cost and complexities compared to TCSC
SVC	Shunt-connected with various possible configurations of thyristor-controlled capacitors and reactors	Total susceptance is controlled by controlling the firing angle of the thyristor. This in turn controls the terminal voltage of the connected bus.	• Cheaper than STATCOM, with lower losses • Slower response due to time delay associated with thyristor-switching
STATCOM	Shunt-connected, employs voltage source converter with pulse width modulation controller	The voltage source converter converts a DC voltage into sinusoidal output voltage with controllable amplitude and phase angle in order to provide reactive power compensation for the connected power system.	• Offers better performance than SCV and exhibits constant current characteristics at low voltages, thus able to inject or absorb reactive power during low voltage grid condition. • Higher losses and higher cost than SVC of similar ratings.
UPFC	Series-shunt connected, combination of series and shunt voltage-source inverters connected via a DC link	Provides active and reactive power flow control by means of the series and shunt converters operating via a common DC link and shunt capacitor storage system.	• Combines the advantages of SSSC and STATCOM. • Able to inject and absorb both real and reactive power • Higher cost and complexities than other FACTS type.

## 4 Review of FACTS devices applications for voltage stability enhancement of RE-integrated power grid

This section provides comprehensive review of FACTS applications to improve voltage stability of RE grid.

### 4.1 Voltage stability with wind energy integration and FACTS applications

Since Wind Energy Conversion Systems constitute the largest variable renewable energy sources with large scale integration, several studies have been carried out to investigate voltage stability of power grids with high penetration of wind energy systems [1, 9, 23, 31, 32, 33, 34, 35, 36, 37, 38, 39, 40, 41, 42, 43, 44, 45, 46, 47, 48]. Three of the most-commonly investigated WECS are the Permanent Magnet Synchronous Generator (PMSG) based WECS, Doubly-Fed Induction Generator (DFIG) and Squirrel-Cage Induction Generator (SCIG). When the SCIG-WECS is incorporated into a power system, it does not have the ability to generate reactive power into the system at the point of common coupling, rather it absorbs reactive power from the grid. This is why a suitably-sized capacitor bank is connected across its stator terminals in order to provide reactive power support at the point of common coupling. However, DFIG-WECS can both generate and absorb reactive power in order to regulate the terminal voltage of the connected bus. This implies that voltage stability of the grid is more adversely affected by SCIG-WECS than DFIG-WECS.

The authors of reference [32] carried out a comparative study on the voltage stability of a power system integrated with SCIG-WECS and DFIG-WECS. PV curves were used to show the maximum loading limits when each WECS type was connected. Two FACTS devices, namely, SVC and STATCOM were also employed for reactive power compensation with their performances compared on a modified IEEE 14-bus test system. The results showed that STATCOM provides a better voltage support than SVC. Also, the reactive power control performance of STATCOM and SVC with DFIG-WECS under short circuit fault was compared in [34]. The simulation results also showed that STATCOM provides better performance than SVC.

In [36], static and dynamic voltage stability analyses of grid-tied wind farms incorporating FACTS devices were carried out. The static analysis was done using static techniques such as power flow, PV curve analysis, and QV modal analysis to examine the voltage stability of IEEE 14-bus test system while the dynamic analysis was performed to evaluate the performance of SVC and STATCOM during both normal and contingency conditions. The results show that both SVC and STATCOM can improve the steady-state voltage stability and the network loadability margin, however, STATCOM was shown to provide a better enhancement for the dynamic voltage stability enhancement.

Furthermore, in [42], the authors investigated the stability improvement of power systems connected with SCIG-WECS, DFIG-WECS, and a combined wind farm comprising both SCIG and DFIG WECS. The SCIG-WECS and DFIG-WECS were equipped with SSSC controller, while the combined wind farm was without any FACTS device. Voltage Stability Index (VSI) was used to examine the voltage stability of the three scenarios. The results of their study showed that although the SSSC enhanced the performance of the SCIG-WECS and DFIG-WECS scenarios, the combined wind farm without the SSSC controller had the best performance. However, the authors of this work only considered SSSC. They did not go further to investigate the performance of other FACTS devices.

Several analytical tools and procedures have evolved to study voltage stability. These include continuation power flow and eigenvalue analysis. For instance, in [49], CPF method and eigenvalue analysis was used to assess the voltage stability of Kerala grid, which is a 220KV, 26-bus system with wind power integration and SVC application.

The studies above show that STATCOM provides better performance than SVC when it comes to voltage stability enhancement.

### 4.2 Voltage stability with solar photovoltaic (SPV) integration and FACTS applications

Several studies have also been carried out on the effects of SPV integration on the voltage stability of a power system [50, 51, 52, 53, 54, 55, 56, 57, 58, 59, 60, 61, 62, 63, 64, 65]. The impact of solar photovoltaic system on the dynamic voltage stability of a power system has been examined in [51]. The authors used Dominion Virginia Power system for the study, with different PV penetration scenarios to illustrate the influence of SPV penetration on the dynamic voltage stability of the system. The results showed that the dynamic voltage stability of a power grid is significantly affected when there is high SPV penetration.

A new way to utilize SPV inverter as STATCOM has been presented in [53]. This device is called PV-STATCOM and it can be used to improve day and night power transmission limits. Studies on transient stability were performed using a realistic single-machine infinite-bus power system, with PV-STATCOM situated at the midpoint. The analysis was performed using Electromagnetic Transients with Direct Current/Power System Computer Aided Design (EMTDC/PSCAD) software. The results of the study showed that the PV-STATCOM can appreciably increase the stable power transmission limits during the night and during the day even with large active power generation.

Also, in [57], the authors investigated the use of PV-STATCOM with voltage and damping controllers to improve the transmission line power transfer capacity. Harmonic analysis was also performed, with the PV-STATCOM located at the centre of the transmission line. The results of the work showed that the PV-STATCOM is effective for system stability improvement by reduction in Total Harmonic Distortion (THD) during fault conditions.

Furthermore [64], explores the possibility of optimizing additional reactive power and active power reduction control strategies for SPV plants. PSO technique was utilised for tuning the real and reactive power support. The optimization technique utilised a combination of the rates of change of frequency, rates of change of voltage and rates of change of voltage phase angle. The simulation results showed that active and reactive power support of SPVs has an undesirable effect on grid stability after the occurrence of a fault, but that the PSO-based tuning of the SPV parameters can mitigate this negative effect.

### 4.3 Optimization and control of FACTS devices for RE-integrated power grid

For many practical applications, FACTS devices need to be optimally selected, sized, and located. They also need to be optimally tuned and controlled for effective performance under changing grid operating conditions. Several works have been done in this regard. In [66], an optimal STATCOM controller has been proposed in order to enhance wind-integrated power grid under fault conditions. Ant Colony Optimization (ACO) and Particle Swarm Optimization (PSO) techniques were employed to obtain a flexible PI parameter tuning necessary for the improvement of STATCOM's dynamic behaviour during a voltage sag. The proposed methodology was tested on a 9 MW DFIG-WECS integrated to 120kV power grid. The impacts of deep voltage sag on this system was also investigated. The findings illustrated the effectiveness of the proposed methods. Also, PSO technique was applied in [67] to design SVC and TCSC coordinated parameters in order to achieve voltage profile improvement. The method was evaluated on IEEE 9-bus system using MATLAB. A multi-objective voltage stability control strategy using SVC and TCSC was also carried out in [68].

New control algorithms are being developed and applied for voltage control of power grid. One of these is the Model Predictive Control (MPC). Few studies have investigated the applications of MPC for voltage stability of power grids. Several of these studies focus on the application of MPC for load control [8, 69, 70, 71, 72]. However, applications of neural-based predictive control (NPC) to control FACTS devices was investigated in [73]. The NPC was used to control bus voltages using STATCOM and real power flow through the use of SSSC. The design and performance of the NPC was compared with the conventional Proportional-Integral (PI) controller particularly in terms of overshoots and quality of control signals. The results indicate that the NPC is a convenient tool for the implementation of adaptive control of a power system.

Also, a comparative study of Deadbeat Controller and Model Predictive Controller applied to Distribution-STATCOM (DSTATCOM) for power quality improvement has been carried out in [74]. While the state-space model of the system was utilised in deadbeat predictive algorithm to compute the required reference value of current so as to obtain the desired value for load current, a discrete-time model of the system was employed in MPC to predict the future current behaviour for each possible voltage vector obtained from the DSTATCOM, after which the voltage vector that minimises a cost function was chosen and applied. MATLAB-Simulink model was utilised to examine the effectiveness of the two controllers. The controllers have been shown to improve the performance of DSTATCOM in achieving voltage control, harmonic mitigation, power factor correction and load balancing, thereby resolving the issues of power quality.

The design of Multiple Input Multiple Output Nonlinear Optimal Predictive Control (NLOPC) system for UPFC control has been proposed in [75]. This control strategy was applied to the *dq* mathematical models of the shunt and series UPFC components together with the dynamic voltage model of the DC link. A one-machine, two-line infinite-bus system with UPFC installed was employed to assess the robustness of the proposed strategy via simulations performed in EMTDC/PSCAD software environment. The results demonstrated the effectiveness of the control scheme over the conventional PI controller in providing closed loop stability for the system and a satisfactory tracking behaviour as well. A similar NLOPC scheme has been applied to STATCOM in [76]. The simulation results also demonstrated that NLOPC can effectively damp low frequency oscillations, maintain transient stability and enhance dynamic performances of a power system with STATCOM installed.

However, these works did not clearly depict the effectiveness of NLOPC scheme to enhance voltage stability of RE-integrated grid under different grid operating conditions. In addition, the works considered only single-machine, double-line infinite bus systems. Therefore, further analyses need to be carried out to provide practical insights for a real power system application.

## 5 Future research direction on FACTS device application for voltage stability improvement of power system with high renewable energy share

This section discusses the future research direction and potential areas that need to be further investigated on the employment of FACTS devices for improved voltage stability of power system with increased renewable energy integration.

### 5.1 Research into robust and efficient ancillary services for increasing renewable energy penetration level

Considering the possibility of 100% or close to 100% renewable energy penetration level in the near future, there is a need to further investigate and thoroughly analyse the required ancillary services such as voltage control, reactive power compensation and power quality enhancement. This becomes necessary for such future grids to be technologically viable and economically sustainable. More efficient and robust FACTS devices will also need to be designed, developed and deployed for power system stability enhancements of such grids. In addition, the associated power quality issues inherent with the high penetration of power electronic-based devices will also need to be addressed.

### 5.2 Consideration of more renewable energy sources

There is paucity of studies that addresses ways of improving the voltage stability of power systems using FACTS devices when more than one RE generation sources are utilised in the system. Most works focus on either grid-connected WECS or grid-integrated solar PV systems. Therefore, more comprehensive analysis is required to study the voltage stability of renewable-integrated grid with two or more RE sources.

### 5.3 Cost and performance trade-off

Studies have shown the different performance level of each FACTS devices on voltage stability enhancement. For instance, STATCOM has been shown to provide better voltage support than SVC, however, it costs more than SVC of the same rating. Thus, SVC may be a cheaper alternative for developing economies than STATCOM. Therefore, a satisfactory trade-off between cost and performance will be required in order to arrive at the most optimal and cost-effective FACTS device to be selected for specific grid applications. This trade-off scenarios needs to be further investigated particularly for evolving power systems such as those found in Sub-Sahara Africa.

### 5.4 Further research on PV-STATCOM

The afore-mentioned studies on solar PV integration have illustrated the potential application of PV-STATCOM for voltage stability enhancement. A more comprehensive work on the practical usefulness and implementation of PV-STATCOM needs to be further investigated with appropriate control strategies suitable for the evolving modern power grid.

### 5.5 Potential application of energy storage system to improve voltage stability

Recently, some studies are focusing on the possibility of utilizing energy storage devices to enhance short-term voltage stability [77, 78, 79]. Some of the devices being investigated include superconducting magnet energy storage and battery energy storage system. However, there is paucity of research work in this aspect when there is increasing renewable energy penetration. Therefore, further investigations on the applications of energy storage systems for voltage stability needs to be carried out, in view of the present trend of increasing RE penetration.

## 6 Conclusion

This paper has presented a comprehensive review on the use of FACTS devices to enhance voltage stability of RE-integrated power grid. An overview on the concept of voltage stability and FACTS devices have also been presented. A representative emphasis was placed on SVC and STATCOM devices.

In addition, this paper has discussed several works that have been carried out on voltage stability improvement of RE-integrated power grids, with specific emphasis on WECS and Solar PV systems. Potential areas for further research have also been presented and discussed. FACTS devices are expected to continue to play crucial roles in voltage stability improvement for the evolving modern power grid, which is to be characterized by increasing renewable energy penetration.

## Declarations

### Author contribution statement

All authors listed have significantly contributed to the development and the writing of this article.

### Funding statement

This work was supported by the 10.13039/501100015776African Union Commission.

### Data availability statement

No data was used for the research described in the article.

### Declaration of interests statement

The authors declare no conflict of interest.

### Additional information

No additional information is available for this paper.
